# A New Binning Method for Metagenomics by One-Dimensional Cellular Automata

**DOI:** 10.1155/2015/197895

**Published:** 2015-10-18

**Authors:** Ying-Chih Lin

**Affiliations:** ^1^Masters Program in Biomedical Informatics and Biomedical Engineering, Feng Chia University, No. 100, Wenhwa Road, Seatwen, Taichung 40724, Taiwan; ^2^Department of Applied Mathematics, Feng Chia University, No. 100, Wenhwa Road, Seatwen, Taichung 40724, Taiwan

## Abstract

More and more developed and inexpensive next-generation sequencing (NGS) technologies allow us to extract vast sequence data from a sample containing multiple species. Characterizing the taxonomic diversity for the planet-size data plays an important role in the metagenomic studies, while a crucial step for doing the study is the *binning* process to group sequence reads from similar species or taxonomic classes. The metagenomic binning remains a challenge work because of not only the various read noises but also the tremendous data volume. In this work, we propose an unsupervised binning method for NGS reads based on the one-dimensional cellular automaton (1D-CA). Our binning method facilities to reduce the memory usage because 1D-CA costs only linear space. Experiments on synthetic dataset exhibit that our method is helpful to identify species of lower abundance compared to the proposed tool.

## 1. Introduction

With the rapid development of next-generation sequencing (NGS) technologies, the ability to gain experimental data has far surpassed the capability to proceed with further analysis. High-throughput NGS machine is capable of sequencing millions to even billions of reads (short DNA fragments) in parallel from a sample containing many species. Within a reasonable cost, an individual laboratory can generate terabase scales of sequencing data within a day [1], which also inspires many mining tools to interpret these data [2]. Instead of traditional works for studying microbial genome on an individual bacterial strain, NGS technologies as a powerful tool greatly facilitates researchers to study the genomes of multiple microorganisms from environmental samples, while it is known as* metagenomics*. Several metagenomic projects have successfully offered valuable insights to the diverse microbial communities, such as the soil [3] and human gut [4].

An important step in metagenomic analysis is the* binning* procedure to keep together reads from similar species or taxonomic classes. There are two major methodologies for binning algorithm: supervised and unsupervised methods [5]. The former is taxonomy-dependent and similarity-based where individual reads are taxonomically grouped by aligning them to known genomes in reference databases, and subsequently reads aligned to similar genomes are grouped into bins. However, in a typical metagenomic scenario, most reads (up to 99% [6]) come from genomes of hitherto unknown organisms, which are then nonexistent in current reference databases. Taxonomy-dependent binning methods fail to identify such reads, and generally categorize them as unassigned. One alternative approach is to align the taxonomic marker genes, for example, recA, rpoB, and 16S ribosomal RNA (rRNA) [7], or particular genomic regions, for example, the internal transcribed spacer (ITS) regions [8].

As for the unsupervised method, it is taxonomy-independent and groups reads from the dataset based on the genomic signatures, such as *k*-mer distribution, G + C content, and codon usage [5, 9], which can be directly extracted from the nucleotide sequences. According to different signatures or observations, a number of composition-based methods are proposed as the binning tools. AbundanceBin [10] utilizes the *k*-mer frequency to group reads, while TOSS [11] is based on sufficiently long mers and integrates AbundanceBin into separating reads from species with different abundances. Both fail to tackle reads from different species with similar abundance ratio [12]. The series of unsupervised binning tools of MetaCluster [12] are developed according to multiple observations, and MetaCluster 5.0 can compute the number of species shaped by the sequence reads. However, it often gives inaccurate number of species for the relatively large number of species in the dataset from the performance comparison to the binning tool MCluster [13].

On the other hand, a* cellular automaton* (CA) is a discrete computational model studied for the complex systems in mathematics, computer science, economics, biology, and so forth. It consists of a regular array of cells, with each being a finite state automaton (FSA), while the array can be in a positive number of dimensions. The state of a cell at time *t* is a function of the states of its neighboring cells at time *t* − 1, where the function is a set of* transition rules*. One-dimensional CA considers the cells over a one-dimensional array and has been used for solving synchronization problems [14], prime generation [15], data clustering [16], real-time language recognition [17], and so on. In this work, we propose a new binning approach for NGS reads from metagenomic sequences based on one-dimensional CA by the extension of previous work [16]. Since a one-dimensional CA requires only linear memory space when running, our binning method moderates the tremendous amount of memory usage caused by NGS data. In addition, we conduct experiments to evaluate the performance and compare it with the proposed tool.

This paper is organized as follows.  Section 2 introduces one-dimensional CA and our binning method step by step. Subsequently, we take the simulated dataset to assess the performance in Section 3. Finally, Section 4 draws our conclusion.

## 2. Binning by One-Dimensional Cellular Automaton

### 2.1. One-Dimensional Cellular Automaton

Cellular automata are discrete models for dynamic systems, where it was originally introduced as a computational medium for machine self-replication guided by a set of rules. The classical version of CA is based on the use of a regular array, local variables, and a function working over a neighborhood. More formally, the regular grid of CA is a set of locally interconnected FSAs that is typically placed over a regular *d*-dimensional lattice *ℒ* [18]. Take the two-dimensional CA as an example, it consists of a lattice of *ℒ* = *m* × *n* squares called* cells*, where each is in one of a finite number of states. The* neighborhood* of a cell *C* is a set of topologically neighboring cells around *C*, and a* transition rule ϕ* applied to *C* defines the change of each cell in the neighborhood of *C* from its current state to a new one. At each iteration, the transition rule is performed on all cells. Though the number of CA applications to engineering problems is relatively few, CA has been largely involved in the simulations of complex systems [18].

We introduce herein a mathematical model based on the one-dimensional CA, whose cells are placed over a linear lattice *ℒ*, to describe the binning procedure for metagenomic sequences. The number of cells in the discrete lattice *ℒ* equals the number of data items *N* in the dataset. At the *t*th iteration, each cell *c*_*i*_(*t*) for *i* ∈ *ℒ* is the *i*th cell of *ℒ* and associates with a specific item in the dataset. For a particular *r* ∈ {3,4,…, *R*}, applying the transition rule *ϕ*_*r*_ to the cell, *c*_*i*_ updates the neighborhood of *c*_*i*_ within the range *r*, where the parameter *R* can be calculated from the number of cells *N*. A greater value of *R* allows a greater size of cluster. Moreover, the boundary condition of one-dimensional CA can be periodic, fixed, or reflecting among others [18]. Here, we set the periodic manner to simulate a circular boundary; that is, *c*_*N*+1_ = *c*_1_.

### 2.2. Transition Rules

In the beginning of *t* = 0, the data item is randomly assigned to a cell in *ℒ*, and then each cell evolves according to the function of its current state and neighboring cells, which is identified by the transition rule *ϕ*_*r*_ for *r* ∈ {3,4,…, *R*}. The value of *r* starts from 3 due to the minimum requirement of three neighboring cells. We say that an iteration is finished if all transition rules are performed on each cell in *ℒ*. There are two common terminated criteria to the whole process: one is the user-defined value for the maximum number of iterations and the other is the convergence of *ℒ* to a stable state; that is, *c*_*i*_(*t*) = *c*_*i*_(*t* − 1), ∀*i* = 1,2,…, *N*. We adopt the latter criterion in this work. In other words, our algorithm is terminated when there is no state change between two consecutive iterations.

**Figure 1:**
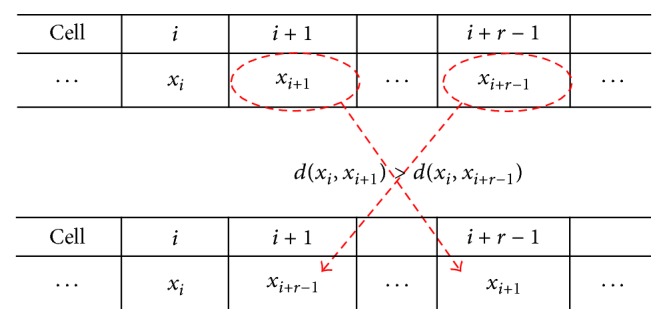
Data items *x*_*i*_, *x*_*i*+1_, and *x*_*i*+*r*−1_ are at cells *c*_*i*_, *c*_*i*+1_, and *c*_*i*+*r*−1_, respectively. The transition rule *ϕ*_*r*_ applied to *c*_*i*_ exchanges *x*_*i*+1_ and *x*_*i*+*r*−1_ if *d*(*x*_*i*_, *x*_*i*+1_) > *d*(*x*_*i*_, *x*_*i*+*r*−1_).

At the *t*th iteration, let *x*_*i*_ be the data item at the cell *c*_*i*_ of *ℒ*. The transition rule *ϕ*_*r*_ to the cell *c*_*i*_ concentrates on three items of *x*_*i*_, *x*_*i*+1_ and *x*_*i*+*r*−1_ by comparing their distances, which is the relation measurement between two items. Let *d*(*x*_*i*_, *x*_*j*_) be the* distance* between two data items *x*_*i*_ and *x*_*j*_. The rule *ϕ*_*r*_ applied to *c*_*i*_ swaps *x*_*i*+1_ and *x*_*i*+*r*−1_, provided that *d*(*x*_*i*_, *x*_*i*+1_) > *d*(*x*_*i*_, *x*_*i*+*r*−1_), illustrated by Figure 1; otherwise, *ϕ*_*r*_ leaves the data items on *c*_*i*+1_ and *c*_*i*+*r*−1_ unchanged. For example, the rule *ϕ*_5_ compares the distance *d*(*x*_1_, *x*_2_) with *d*(*x*_1_, *x*_5_) when applying it on *c*_1_, with *d*(*x*_1_, *x*_6_) on *c*_2_ and so on. When *ϕ*_5_ is applied on the cell *c*_*N*−3_, it considers the distances *d*(*x*_*N*−3_, *x*_*N*−2_) and *d*(*x*_*N*−3_, *x*_1_), due to the periodic boundary condition.

To compute *d*(*x*_*i*_, *x*_*j*_), two feature vectors ${\vec{x}}_{i}$ and ${\vec{x}}_{j}$ are extracted, and then we set $d(x_{i},x_{j})=\left\|{\vec{x}}_{i}-{\vec{x}}_{j}\right\|$. Determining the features of data is of great importance as it directly affects the measurement among data items. Typically, ideal features should be useful in distinguishing homologous patterns from the dataset, immune to noises as well as easy to extract and interpret. Such an elegant selection of features can greatly reduce the workload and simplify the subsequent clustering process. For metagenomic data, composition-based binning methods have employed several features to assign reads to different groups, where these features can be directly extracted from the nucleotide sequences including the *k*-mer frequency, G + C content, and codon usage [5]. In this work, we select the *k*-mer spectrum of a read as its feature. Previous studies suggest that *k* = 4 or 5 is the better choice to extract features from metagenomic sequences [12, 19]. As a result, we take *k* = 5 to represent each read as a 4^5^-dimensional vector, and, by the transition rules, sequence reads with similar feature vectors are assembled. When the one-dimensional CA converges after a number of iterations, the reads within the same cluster would be arranged in sequence at the lattice.

### 2.3. Boundary Detection

The transition rules arrange the data with similar feature vectors in a chain of cells, while a problem emerges from the chain order of data: how to group the consecutive data over *ℒ* as clusters. A feasible strategy is to identify the* boundary point*, which is the data object located in the edge of a cluster and thus may have multiple cluster features. Boundary detection of clusters plays an important role in applications, such as image processing and machine learning, but its study is still in the infancy since the first work [20]. As usual, it is challenging to accurately recognize boundary points in an efficient way due to the interference of noise and outlier points.

**Figure 2:**
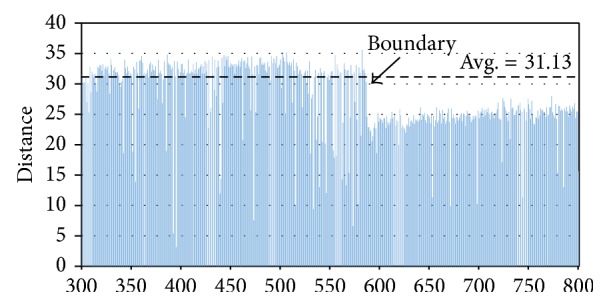
Chainmap diagram to our simulation result, where only the distances of cell numbers from 300 to 800 in the final state are shown. The average distance of whole dataset is 31.13, while the ideal boundary is marked at the cell *c*_586_.

We detect the boundary points according to the partial order over *ℒ* given by the one-dimensional CA.  Figure 2 is the chainmap diagram of our simulation result, in which only the cell numbers from 300 to 800 in the final stationary state are shown. This diagram is composed of the distances between two items at consecutive cells, and generally the low distances of consecutive cells imply that the corresponding items should be in the same cluster. Conversely, the cells with sharp variation on distances could be candidates for the cluster boundary. It seems as if the challenge to detect boundary here can be solved by the basic sequential clustering scheme (BSAS) [21], but they are slightly different. First, the data items are arranged in a particular order retrieved from the convergence result of one-dimensional CA; second, the user-specified parameters, including the threshold of dissimilarity and maximum allowable number of clusters, as in the conventional BSAS, are not required.

It looks like there are two different clusters in Figure 2 because the distance distribution is not consistent in these cells, which inspires us to develop a straightforward way for detecting cluster boundaries. In the final state of one-dimensional CA, we consider the data item *x*_*i*_ at *c*_*i*_. Let avg_*k*_^*i*^ and sd_*k*_^*i*^ be the average and standard deviation, respectively, from the starting item of the cluster *k* to the item *x*_*i*_ at *c*_*i*_. Also, the average distance of the whole dataset is denoted as avg. Therefore, a item *x*_*i*_ is regarded as a starting point of a new cluster if the following three conditions are satisfied: (1) *d*(*x*_*i*_, *x*_*i*+1_) < avg; (2) *d*(*x*_*i*−1_, *x*_*i*_) > max⁡(avg, avg_*k*_^*i*−1^ + sd_*k*_^*i*−1^), where *x*_*i*−1_ belongs to the cluster *k*; (3) *d*(*x*_*i*−1_, *x*_*i*_) > *d*(*x*_*i*_, *x*_*i*+1_) > *d*(*x*_*i*+1_, *x*_*i*+2_). The first criterion asks that the starting point of a new cluster should have a smaller distance than the average; the second one requests the distance between two consecutive items of a cluster boundary, that is, one is the end and the other is the start of two distinct clusters, respectively, to be bigger enough; the final criterion emphasizes the relations among consecutive items near the boundary, which is helpful to filter the noise. We use the three criteria to determine whether *x*_*i*_ is the starting point of a new cluster, and if yes, the item *x*_*i*−1_ is also the boundary of the preceding cluster.

### 2.4. Binning Method

The binning approach takes the sequencing reads from metagenomic sequences as the input, and its output is to cluster homogeneous reads together as accurate as possible. At first, our binning method assigns to each read a cell of the lattice *ℒ* at random. Then, the one-dimensional CA with the transition rules introduced in Section 2.2 is invoked to move reads accordingly. After a number of iterations, the system converges to a stable state where all reads associated with similar feature vectors are continuously placed on *ℒ*. As long as CA arranges the reads in the chain order by composition-based comparison, the next step is to divide the chain into different slices, that is, clusters. The boundary detection given in Section 2.3 shows three criteria so as to find out the cluster boundaries. In some cases, the second condition of the three criteria for boundary detection is easy to achieve because of the small standard deviation for few items in a new cluster, resulting in excessive noises. Therefore, the value 30 is used to be the minimum size of cluster as a* rule of thumb* [22]. In other words, if *x*_*i*_ at the cell *c*_*i*_ is the starting item of a cluster, then the item located beyond the (*i* + 30)th cell can be the boundary candidate of this cluster. By this way, reads in the chain order can be divided into distinct clusters more correctly.

## 3. Experimental Results

Though there is no generally acknowledged benchmark for binning NGS reads at present, several tools are recently proposed for simulating metagenomics, such as ART [23], NeSSM [24], BEAR [25], and MetaSim [26]. Our binning method is based on the one-dimensional CA (1D-CA) to automatically determine the clusters for reads, and to validate it, the dataset as the work in [13] is generated using MetaSim. The synthetic dataset D9 in [13] consists of three species with the average length of 1,000 bp and the read number of 5,000 as well as the abundance ratio of 1 : 3 : 9. The three species in D9 are *Pseudomonas*_*aeruginosa*_*PAO*1, *Legionella*_*pneumophila*_*str*._*Lens*, and *Cycloclasticus*_*sp*._*P*1. In addition, we run each experiment ten times and compare the result with MCluster [13].

**Figure 3:**
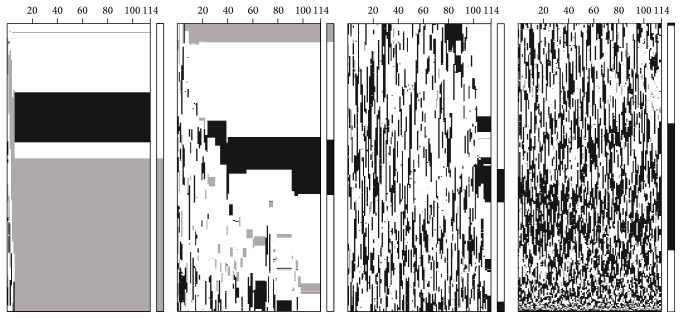
Experimental result of our binning method, where each row in a subdiagram represents the successive states of the corresponding cell and each column shows the cell's state in an iteration. There are totally 5,000 rows (or cells) divided into four parts, and the 1D-CA converges after 114 iterations. There are three colors associated with three species of dataset, and the rightmost bars in the four subdiagrams are the output of our binning method.

Figure 3 exhibits the successive states of 1D-CA. This is a grayscale image with three colors, namely, white, gray, and black, assigned to three species, respectively: white corresponds to the species of the greatest abundance, gray corresponds to the species of the smallest abundance, and black corresponds to the remained one. There are 144 iterations in total, and due to the great amount of reads, we quarter the strip of diagram as shown in Figure 3. In the beginning of the leftmost columns of the four subdiagrams, all reads are put on the lattice in a random order, leading to the mixed colors. At the 6th iteration, the leftmost subdiagram shows two distinct blocks, while the number of blocks becomes four at the end of this subdiagram. From the rightmost subdiagram in Figure 3, we can see that the reads located here are hard to be stable and thus the color distribution is in a state of utter chaos. Moreover, the rightmost bars of the four subdiagrams in Figure 3 are the clustering result of our binning method, in which it fails to identify the clear black block in the leftmost subdiagram.

In addition, we run MCluster on the same dataset and compare its performance with our method. MCluster is developed as the unsupervised method for binning metagenomic sequences, and it has been shown to be better than several works in the overall performance [13]. We run MCluster ten times as it is identical to ours and compute the average of these simulated results. To evaluate the experimental results, we consider two performance metrics,* precision* and FP* rate*. Assume that there are *N*_*s*_ species in the dataset and a binning algorithm identifies *K* clusters *C*_1_, *C*_2_,…, *C*_*K*_. Let *R*_*ij*_ be the number of reads in *C*_*i*_ that are from species *j*, and the cluster *C*_*i*_ is identified as species *s* if arg_*j*_max⁡(*R*_*ij*_) = *s*. The precision can then be defined as follows [12, 13]:

(1) $$\begin{array}{c} \text{precision}=\frac{\sum_{i=1}^{K}\text{ma}{\text{x}}_{j}\left(R_{ij}\right)}{\sum_{i=1}^{K}\sum_{j=1}^{N_{s}}R_{ij}}. \end{array}$$

Since there is no “unclassified reads” in our binning method, two metrics* sensitivity* and* F-measure* are identical to the precision as in [13]; thus, it is excluded here. Moreover, the other metric FP* rate* is to measure the number of reads assigned to incorrect species. Let *ℂ*_*s*_ be the index set of clusters recognized as species *s*. As a result, the FP* rate* of a species *s*, denoted as FP_*r*_(*s*), is defined by

(2) FPrs=∑i∈Cs∑jRij ∣ j=1,2,…,s−1,s+1,…,Ns∑i∈Cs∑jRij ∣ j=1,2,…,Ns.

Given the species *s*, FP_*r*_(*s*) measures the false positive condition to all clusters identified as *s* by the binning method.  Table 1 summarizes the average performances of the simulated result for MCluster and ours. From this table, the 1D-CA precision is significantly lower than the MCluster precision; even so, 1D-CA has something to recommend it. To evaluate the FP* rate*, we denote the species of dataset as I, II, and III and sort them in descending order according to their abundance ratios; that is, the most abundant species corresponds to the symbol I.  Table 1 shows that FP_*r*_(*I*) of 1D-CA is far worse than that of MCluster, implying that 1D-CA is not good at identifying abundant specsies, which is contrary to most works [10–12]. This may be caused by the insensitivity of 1D-CA to detect the cluster boundaries, and hence many reads are inaccurately recognized to the cluster of species I. However, 1D-CA has the best performance in identifying species of lower abundances as shown in Table 1, which helps in extracting rare species from samples and progressing the assembly work with ease.

## 4. Conclusions

Recent technologies on high-throughput NGS grow rapidly such that it is easy and cheap to sequence all individuals of a microbial community from environmental samples. Metagenomic analysis parses the vast amount of sequence data to mining valuable insights, where the binning process is a crucial step to group sequence reads from similar species or taxonomic classes. Due to the read noises and planet-size data, the binning step is challenging in metagenomic studies. In this paper, we propose a new binning method based on the one-dimensional cellular automaton (1D-CA), where 1D-CA has been used for solving synchronization problems, data clustering, language recognition, and so forth. Since 1D-CA requires only linear space when running, our method moderates the tremendous amount of memory usage resulted from NGS reads. Moreover, experiments on synthetic dataset show that our method is helpful to identify species of lower abundance compared to the proposed tool, which facilitates the recognition of rare species from environmental samples.

## Figures and Tables

**Table 1:** Performance comparison.

	1D-CA			MCluster [13]		
Precision	0.754			0.842		

*s*	I	II	III	I	II	III

FP_*r*_(*s*)	0.262	0.317	0.004	0.031	0.356	0.006
